# The pretreatment lymphocyte to monocyte ratio predicts clinical outcome for patients with hepatocellular carcinoma: A meta-analysis

**DOI:** 10.1038/srep46601

**Published:** 2017-04-18

**Authors:** Wei Song, Chuan Tian, Kai Wang, Run-jin Zhang, Shu-bing Zou

**Affiliations:** 1 Department of Hepatobiliary Surgery, The Second Affiliated Hospital of Nanchang University, No.1 Minde Road, Nanchang, China; 2 Department of Nuclear Medicine, Guizhou Provincial People’s Hospital, Guiyang, China

## Abstract

The lymphocyte-to-monocyte ratio (LMR) has been reported to predict clinical outcomes in multiple malignancies. The aim of this study was to assess the prognostic role of pretreatment LMR in hepatocellular carcinoma (HCC). A total of seven studies comprising 2,738 patients were included in the meta-analysis. Pooled results showed that elevated LMR was significantly associated with increased overall survival (OS) (HR: 0.31, 95% CI: 0.20–0.47, p < 0.001), disease-free survival (DFS)/recurrence-free survival (RFS) (HR: 0.57, 95% CI: 0.49–0.67, p < 0.001). The favorable prognostic impact of high LMR on OS was observed in all subgroup with different sample size, type of publication, NOS score, and the cut-off value of LMR. In addition, low LMR was significantly correlated with TNM stage and BCLC stage. We therefore conclude that elevated pretreatment LMR could be a favorable prognostic factor for clinical outcomes in patients with HCC.

According to the American Cancer Society, hepatocellular carcinoma (HCC) is the fifth most common malignancy and the second cause of cancer-related mortality in the United States in 2015^1^,^2^. Around the world, there were an estimated 500,000 to 1 million people died of HCC per year^3^. Liver resection is the most effective therapy for the majority of patients with resectable HCC, but unfortunately most of patients are not eligible for curative resection at the time of diagnosis^4^. In addition, postoperative recurrence rate for patients with early stage HCC can be high at 50% to 70% after liver resection^5^. Therefore, the overall prognosis of HCC patients remains poor. It is vital to develop novel potential biomarker to select appropriate treatment strategies and predict prognosis.

Systemic inflammatory responses can largely influence the formation and recurrence of HCC^6^,^7^,^8^. Several inflammatory biomarkers, such as neutrophil-to-lymphocyte ratio (NLR), lymphocyte-to-monocyte ratio (LMR), and platelet-to-lymphocyte ratio (PLR) are identified as prognostic indicators in a wide variety of solid tumors^9^,^10^,^11^,^12^. These tests are simple and inexpensive to perform, and they are readily available in daily oncologic practice. LMR is a readily available, routinely measured, and inexpensive inflammatory biomarker, which can be easily applied into in daily oncologic practice. Accumulating evidence shows that lymphocytes are known to play a crucial role in suppressing HCC progression through immunoselection in an immunosuppressive network, which dictates immune responses to tumors^13^. A low lymphocyte count is associated with the systemic inflammatory responses and is known to promote cancer progression through effects upon cell-mediated immunity^14^. Conversely, activated circulating monocytes can secrete multiple proinflammatory cytokines, which are involved in tumor development and progression^15^,^16^. As a consequence high monocyte counts are associated with microvascular invasion and poor prognosis in patients with HCC^17^. Moreover, a high LMR, which is defined as absolute lymphocyte counts divided by monocyte counts, is reported to correlate with favorable prognosis in patients with HCC^18^,^19^.

Nevertheless, there has been no meta-analysis to assess the correlation between pretreatment LMR and the survival of HCC patients. Therefore, we conducted a meta-analysis to assess the effects of pretreatment LMR on OS and DFS/RFS in HCC. In addition, the correlations between LMR and the clinicopathological features was also examined.

## Materials and Methods

### Search strategies

We searched MEDLINE, EMBASE, PubMed, and Cochrane databases from inception up to February 2017. Search terms included “HCC” or “hepatocellular carcinoma” or “liver cancer” or “liver tumor” or “liver neoplasms” or “liver cell carcinoma”, “LMR” or “lymphocyte monocyte ratio” or “lymphocyte to monocyte ratio” or “lymphocyte-to-monocyte ratio” or “lymphocyte-monocyte ratio”, “survival” or “prognostic” or “prognosis” or “recurrence” or “clinical outcome”. The references of eligible studies, relevant systematic reviews and meta-analyses in this field were manually retrieved.

### Inclusion criteria

The criteria for inclusion were listed as follows: (1) the diagnosis of HCC were confirmed by pathology; (2) studies assessing the association of pretreatment LMR with OS, DFS/RFS, or CSS; (3) the cut-off value of LMR was reported; and (4) studies supplied sufficient information for calculating hazard ratio (HR) and 95% confidence interval (CI).

### Exclusion criteria

The exclusion criteria were as follows: (1) reviews, letters, case reports, and comments; (2) reporting insufficient data for calculating an HR and 95% CI; and (3) overlapping or duplicate data.

### Data extraction and Assessment of risk of bias

The two reviewers independently reviewed all eligible studies and extracted data. Any disagreement was resolved by a third reviewer. The following information was captured using data abstraction forms:

Study characteristics included first author’s name, year of publication, study design (prospective or retrospective), type of publication, country, ethnicity, survival analysis methods (multivariate, univariate), and time of follow-up. Patient characteristics included patient ages and genders, number of patients, disease stage, treatment, and cut-off value. Outcome measures included HRs for OS, DFS, RFS, or CSS as well as their 95% CIs. HRs were extracted from multivariate or univariate analyses or estimated from Kaplan-Meier survival curves^20^.

The study quality was evaluated in accordance with the Newcastle-Ottawa Scale (NOS)^21^. This included an assessment of subject selection, comparability of groups, and clinical outcome. A total of nine items were extracted and each item scored 1. The maximum score is 9 and those studies with a NOS score ≥7 were considered as high-quality studies.

### Statistical analysis

The meta-analysis was conducted using RevMan 5.3 software (Cochrane Collaboration, Copenhagen, Denmark). Heterogeneity between studies was estimated using Cochrane’s Q statistic and I^2^ statistic^22^. A p-value < 0.1 for the Q-test or I^2^ > 50% indicated significant heterogeneity. When there was no statistically significant heterogeneity, we used the fixed-effects model for pooling the results; otherwise, the random-effects model was applied. HRs and their 95% CIs were searched in the original articles or extrapolated using methods described by Tierney and Parmar^20^,^23^. The log HR and standard error (SE) were used for aggregation of the survival results^23^. The associations between LMR and clinicopathologic features were expressed as odds ratios (ORs) and its 95% CIs. P < 0.05 was defined as statistically significant. Subgroup analyses were conducted for: the sample size, type of publication, NOS score, and the cut-off value of LMR. Sensitivity analyses were carried out to evaluate result stability excluding each study.

## Results

### Search results

**Figure 1:**
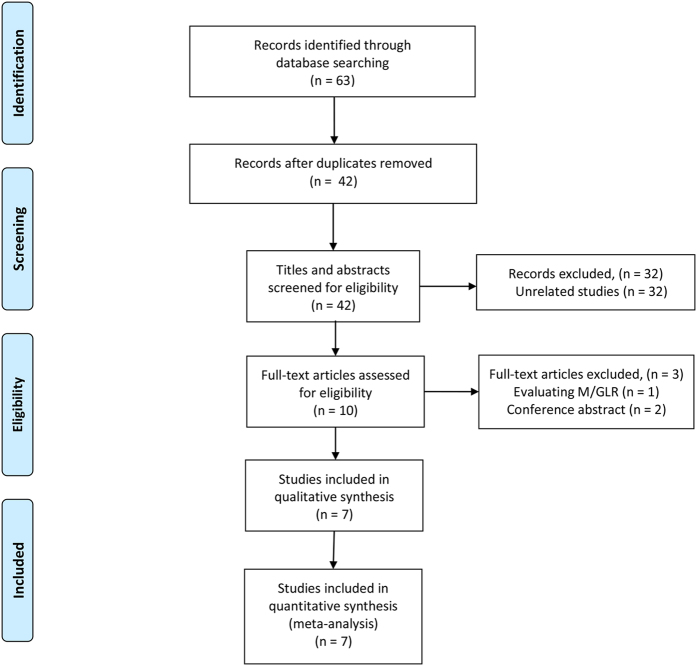
Flow diagram of the study selection process.

Our search strategy yielded 63 potentially relevant records. After duplicates removal (21 records), 42 articles were screened for eligibility. Of these, 32 were excluded through titles and abstracts, leaving 10 articles for further evaluation. Subsequently, 3 articles did not meet the inclusion criteria and were therefore excluded. Eventually, seven studies, comprising a total of 2,507 patients, were considered eligible for the present meta-analysis^18^,^24^,^25^,^26^,^27^,^28^,^29^. The PRISMA flow diagram of the study selection process was shown in Fig. 1.

### Characteristics of the included studies

According to the publication type, 6 and 1 studies were published in full-text and abstract forms, respectively. All included studies were from China and were published between 2014 and 2017. The sample sizes ranged from 208 to 1,020. There were six studies for OS, and all for DFS/RFS. The cut-off values for LMR ranged from 0.83 to 3.77. All included studies consisted of two groups: high and low LMR. HR and 95% CI was extracted directly from the seven studies. In methodological quality of studies, the NOS scores of the included studies ranged from 5 to 8. Detailed patient characteristics and methodological quality are shown in Table 1.

### Meta-analysis

#### LMR and OS in HCC

**Figure 2:**
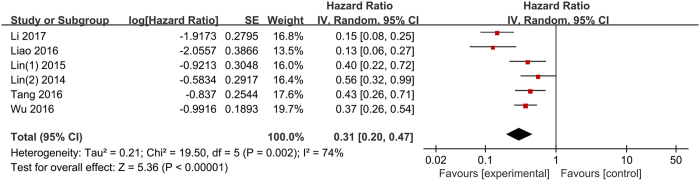
Forest plots for the association between LMR and OS.

Six of the included studies reported the data of LMR and OS in HCC. Overall, elevated LMR had a significant association with increased OS (HR: 0.31, 95% CI: 0.20–0.47, p < 0.001) with significant heterogeneity (p = 0.002, I^2^ = 74%; Fig. 2).

To detect the potential heterogeneity, subgroup analyses stratified by sample size, type of publication, NOS score, and the cut-off value of LMR (Table 2). Subgroup analysis revealed that high LMR predicted increased OS in patient with HCC, regardless of the sample size (≥300 and <300), type of publication (full-text and abstract), NOS score (≥7 and <7), and the cut-off value of LMR (≥3 and <3).

#### LMR and DFS/RFS in HCC

**Figure 3:**
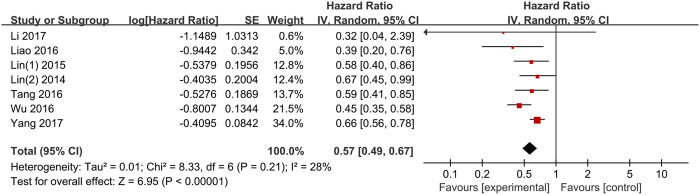
Forest plots for the association between LMR and DFS/RFS.

All included studies reported the data of LMR and DFS/RFS in HCC. A combined analysis demonstrated that elevated LMR was significantly correlated with increased DFS/RFS (HR: 0.57, 95% CI: 0.49–0.67, p < 0.001), with no significant heterogeneity between studies (p = 0.21, I^2^ = 28%; Fig. 3).

#### LMR and clinicopathological features

To further explore the impact of LMR on the clinical features in HCC, we identified 9 clinical factors in HCC. The pooled analysis demonstrated that low LMR was significantly correlated with TNM stage (III-IV vs. I-II; HR = 1.78, 95% CI: 1.19–2.68, P = 0.005) and BCLC stage (B/C vs. A; HR = 1.70, 95% CI: 1.21–2.39, P = 0.002). Whereas no significant association was found with gender, AFP, liver cirrhosis, tumor differentiation, tumor number, tumor size, and vascular invasion. The correlation between LMR expression and clinicopathological parameters of HCC is shown in Table 3.

#### Sensitivity analysis

In order to assess the influence of single studies on the overall estimate, the sensitivity analysis was performed. Each single study was removed each time to estimate the influence of individual data sets on the combined HR for OS. The results showed that no study had a significant effect on the observed effect size (pooled HR), indicating the robustness of our findings.

## Discussion

Hepatocellular carcinoma (HCC) is mainly caused by viral infections such as hepatitis B virals (HBV) and hepatitis C virals (HCV)^3^. Mounting studies indicated that infiltration of inflammatory cells in the tumor microenvironment significantly affected the biological behavior of HCC^25^. In the tumor microenvironment, the inflammatory immune cells have been implicated in HCC progression^30^,^31^. The pretreatment LMR, as a promising inflammatory biomarker, is reportedly linked to prognosis in patients with HCC^18^,^19^,^28^. Therefore, LMR may serve as a promising prognostic factors for HCC patients. To our knowledge, this is the first meta-analysis to investigate the prognostic effect of pretreatment LMR in HCC.

Our meta-analysis provides strong evidence that the presence of high LMR significantly increases OS and DFS/RFS in patients with HCC. Stratified analysis demonstrated that high LMR was significantly correlated with increased OS in patient with HCC, regardless of the sample size, type of publication, NOS score, and the cut-off value of LMR. In addition, we further assessed the correlation between pretreatment LMR and clinicopathological features. The pooled data showed low LMR was linked with TNM stage and BCLC stage. Therefore, pretreatment LMR may serve as a promising prognostic biomarker for estimating HCC prognosis. Furthermore, we also performed sensitivity analysis and found that no study had a significant effect on the observed effect size (pooled HR), indicating the robustness of our findings.

The exact molecular mechanisms responsible for the prognostic impact of LMR in HCC are unclear. It has been suggested that cross-talk between the inflammatory response and tumor progression play a critical role in the initiation and progression of HCC^14^,^32^,^33^. In the tumor microenvironment, inflammatory infiltrates have a large influence on the biological behavior of HCC^34^,^35^. Tumor-infiltrating lymphocytes (TILs), as representative component of the immune microenvironment, are implicated in several stages of HCC progression, and TIL phenotypes may be a predictor for favorable prognosis^36^,^37^. Conversely, low lymphocyte counts might result in an insufficient immunological reaction, which lead to inferior survival in multiple cancers^38^,^39^. In addition, infiltrated CD4+ and CD8+ T cells interaction among each other are essential to the antitumor immune response by inducing tumor cell apoptosis^40^,^41^. Monocytes infiltrating tumor tissue are also involved in HCC development and progression^42^. Activated monocytes in HCC microenvironments can trigger and polarize T-cell responses and faciliate inflammation-induced tumor development^43^. Tumor-associated macrophages (TMAs) are derived from circulating monocytes. TAMs can accelerate HCC cell proliferation, tumor-associated angiogenesis, and metastasis^30^,^37^,^44^. Several studies showed that high infiltration of TAMs predicted decreased survival in various cancers^45^,^46^. Given this background, the observed favorable of high pretreatment LMR on OS of cancer patients may reflect the critical function of LMR in an inflammatory tumor microenvironment that inhibits tumor progression.

Nevertheless, there were several limitations to the present study. First, excessive heterogeneity existed among studies. Subgroup analyses didn’t find the potential sources of heterogeneity. In addition, we performed sensitivity analysis. The results showed that no study had a significant effect on the observed effect size. Second, the cut-off value for LMR was not unified in each study. Third, all included studies were retrospective analysis. Fourth, all included studies were from China, which limited our conclusions for other ethnic populations.

In conclusion, our findings demonstrated that an elevated pretreatment LMR is associated with favorable outcomes in patients with HCC.

## Additional Information

**How to cite this article:** Song, W. *et al*. The pretreatment lymphocyte to monocyte ratio predicts clinical outcome for patients with hepatocellular carcinoma: A meta-analysis. *Sci. Rep.* **7**, 46601; doi: 10.1038/srep46601 (2017).

**Publisher's note:** Springer Nature remains neutral with regard to jurisdictional claims in published maps and institutional affiliations.

## Supplementary Material

Supplementary Information

## Figures and Tables

**Table 1:** Characteristics of the studies included in the meta-analysis.

Author	Year	Country	Type of publication	Follow-up (months)	Treatment	No. of patients	Stage	Cut-off value	Survival analysis	Analysis	NOS score
Li	2017	China	Full-text	33 (6–85)	Surgery	253	Mixed	3.0	OS/RFS	MV/UV	8
Liao	2016	China	Full-text	44 (1.5–84)	Surgery	387	Mixed	3.3	OS/RFS	MV	8
Lin (1)	2015	China	Full-text	34.8 (1.7–106.6)	Surgery	210	Mixed	3.23	OS/RFS	MV/UV	7
Lin (2)	2014	China	Abstract	34 (2–106)	Surgery	210	—	3.2	OS/RFS	MV/UV	—
Tang	2016	China	Full-text	38 (1.5–82.2)	Surgery	208	Mixed	0.83	OS/RFS	MV/UV	5
Wu	2016	China	Full-text	45.5 (2–93)	Surgery	450	Mixed	3.77	OS/RFS	MV/UV	7
Yang	2017	China	Full-text	NA	Surgery	1020	Mixed	3.23	DFS	MV/UV	6

OS: overall survival; DFS: disease-free survival; RFS: recurrence-free survival; MV: multivariate; UV: univariate NA: not available.

**Table 2:** Pooled hazard ratios (HRs) for OS according to subgroup analyses.

Subgroup	No. of studies	No. of patients	Effects model	HR (95% CI)	P value	Heterogeneity	
						I^2^ (%)	P_h_
Overall	6	1718	Random	0.31 (0.20–0.47)	<0.001	74	0.002
Sample size							
≥300	2	837	Random	0.23 (0.08–0.65)	0.005	84	0.01
<300	4	881	Random	0.34 (0.19–0.61)	<0.001	77	0.005
Cut-off for LMR							
≥3	5	1510	Random	0.28 (0.17–0.48)	<0.001	78	0.001
<3	1	208	—	0.43 (0.26–0.71)	0.001	—	—
Type of publication							
Full-text	5	1508	Random	0.27 (0.17–0.44)	<0.001	74	0.004
Abstract	1	210	—	0.35 (0.24–0.50)	0.006	—	—
NOS score							
≥7	4	1300	Random	0.28 (0.17–0.48)	<0.001	78	0.001
<7	1	208	—	0.43 (0.26–0.71)	0.001	—	—

**Table 3:** Meta-analysis of the association between LMR and clinicopathological features of HCC.

Characteristics	No. of studies	No. of patients	OR (95% CI)	p	Heterogeneity	
					I^2^ (%)	Ph
Gender (male vs. female)	3	913	1.16 (0.64–2.09)	0.62	54	0.11
AFP (>400 ng/mL vs. <400 ng/mL)	2	660	1.61 (0.81–3.20)	0.17	73	0.05
Liver cirrhosis (yes vs. no)	3	913	1.66 (0.97–2.83)	0.06	52	0.12
Differentiation (low vs. moderate/high)	3	913	1.04 (0.74–1.46)	0.82	0	0.88
Tumor number (multiple vs. single)	2	660	0.96 (0.62–1.50)	0.87	0	0.61
Tumor size (>5 cm vs. <5 cm)	3	913	1.36 (0.79–2.35)	0.27	69	0.04
TNM stage (III-IV vs. I-II)	2	703	1.78 (1.19–2.68)	0.005	33	0.29
BCLC stage (B/C vs. A)	2	703	1.70 (1.21–2.39)	0.002	0	0.38
Vascular invasion (pos vs. neg)	2	660	1.47 (0.48–4.49)	0.50	84	0.01
