# Supplementary material for: The pretreatment lymphocyte to monocyte ratio predicts clinical outcome for patients with hepatocellular carcinoma: A meta-analysis
Source: Sci Rep. 2017 Apr 18;7:46601. doi: 10.1038/srep46601 (PMC5394547; doi:10.1038/srep46601)
Supplement: Supplementary Information [file srep46601-s1.doc]

**The pretreatment lymphocyte to monocyte ratio predicts clinical outcome for patients with hepatocellular carcinoma: A meta-analysis**

Wei Song^1^,*, Chuan Tian^2^,*, Kai Wang^1^, Run-jin Zhang^1^, Shu-bing Zou^1^

^1^Department of Hepatobiliary Surgery, The Second Affiliated Hospital of Nanchang University, No.1 Minde Road, Nanchang, China

^2^ Department of Nuclear Medicine, Guizhou Provincial People’s Hospital, Guiyang, China

*These authors contributed equally to this work

Corresponding author:

Shu-bing Zou

Email: [zousb999@163.com](mailto:zousb999@163.com,)

Tel: 0791-86291176

Fax: 0791-86291176

**Search strategies**

1. **Chochrane:**

#1 (LMR or lymphocyte to monocyte ratio or lymphocyte monocyte ratio or lymphocyte-to-monocyte ratio or lymphocyte-monocyte ratio)

#2 MeSH descriptor Carcinoma, Hepatocellular explode all trees

#3 (((liver or hepatic or hepatocellular or hepato-cellular) and (carcinom* or cancer* or neoplasm* or malign* or tumo*)) or HCC)

#4 #2 or #3

#5 (survival or prognosis or recurrence or clinical outcome)

#6 #1 and #4 and #5

1. **MEDLIME(Ovid SP):**

1 (LMR or lymphocyte to monocyte ratio or lymphocyte monocyte ratio or lymphocyte-to-monocyte ratio or lymphocyte-monocyte ratio).mp

[2 exp Carcinoma, Hepatocellular/](http://ovidsp.tx.ovid.com.nyu.pubmed007.cn:8080/sp-3.24.1b/ovidweb.cgi?&Controlled+Vocabulary=Mapping|0&Return=mapping&S=GNMKFPNIDGDDAEGENCHKCEGCOMAGAA00)

[3 exp Liver Neoplasms](http://ovidsp.tx.ovid.com.nyu.pubmed007.cn:8080/sp-3.24.1b/ovidweb.cgi?&Controlled+Vocabulary=Mapping|0&Return=mapping&S=GNMKFPNIDGDDAEGENCHKCEGCOMAGAA00)/

4 (((liver or hepatic or hepatocellular or hepato-cellular) and (carcinom* or cancer* or neoplasm* or malign* or tumo*)) or HCC).mp

5 2 or 3 or 4

6 (survival or prognosis or recurrence or clinical outcome).mp

7 1 and 5 and 6

1. **Embase(Ovid SP):**

1 (LMR or lymphocyte to monocyte ratio or lymphocyte monocyte ratio or lymphocyte-to-monocyte ratio or lymphocyte-monocyte ratio).mp

2 exp liver cell carcinoma/

3 exp liver cancer/

4 exp [liver tumor](http://ovidsp.tx.ovid.com.nyu.pubmed007.cn:8080/sp-3.24.1b/ovidweb.cgi?&Controlled+Vocabulary=Mapping|0&Return=mapping&S=NNDNFPDJBGDDAEEPNCHKMFGCCHFOAA00)/

5 (((liver or hepatic or hepatocellular or hepato-cellular) and (carcinom* or cancer* or neoplasm* or malign* or tumo*)) or HCC).mp

6 2 or 3 or 4 or 5

7 (survival or prognosis or recurrence or clinical outcome).mp

8 1 and 6 and 7

1. **Pubmed:**

((((LMR or lymphocyte to monocyte ratio or lymphocyte monocyte ratio or lymphocyte-to-monocyte ratio or lymphocyte-monocyte ratio))) AND (((Hepatocellular Carcinoma[MeSH Terms]) OR Liver Neoplasms[MeSH Terms]) OR (((liver or hepatic or hepatocellular or hepato-cellular) and (carcinom* or cancer* or neoplasm* or malign* or tumo*)) or HCC))) AND ((survival or prognosis or recurrence or clinical outcome))
